# Predicting alveolar nerve injury and the difficulty level of extraction impacted third molars: a systematic review of deep learning approaches

**DOI:** 10.3389/fdmed.2025.1534406

**Published:** 2025-05-20

**Authors:** Hamza Al Salieti, Hanan M. Qasem, Sakhr Alshwayyat, Noor Almasri, Mustafa Alshwayyat, Amira A. Aboali, Farah Alsarayrah, Lina Khasawneh, Mohammed Al-mahdi Al-kurdi

**Affiliations:** ^1^ Faculty of Dentistry, Applied Science Private University, Amman, Jordan; ^2^ Faculty of Dentistry, Jordan University of Science and Technology, Irbid, Jordan; ^3^ Research Associate, King Hussein Cancer Center, Amman, Jordan; ^4^ Internship, Princess Basma Teaching Hospital, Irbid, Jordan; ^5^ Applied Science Research Center, Applied Science Private University, Amman, Jordan; ^6^ Faculty of Medicine, University of Jordan, Amman, Jordan; ^7^ Faculty of Medicine, Jordan University of Science and Technology, Irbid, Jordan; ^8^ Damanhour Teaching Hospital, General Organization for Teaching Hospitals and Institutes, Damanhour, Egypt; ^9^ Department of Prosthodontics, Faculty of Dentistry, Jordan University of Science and Technology, Irbid, Jordan; ^10^ Faculty of Medicine, University of Aleppo, Aleppo, Syrian Arab Republic

**Keywords:** alveolar nerve, deep learning, mandibular nerve, panoramic radiographic, third molar

## Abstract

**Background:**

Third molar extraction, a common dental procedure, often involves complications, such as alveolar nerve injury. Accurate preoperative assessment of the extraction difficulty and nerve injury risk is crucial for better surgical planning and patient outcomes. Recent advancements in deep learning (DL) have shown the potential to enhance the predictive accuracy using panoramic radiographic (PR) images. This systematic review evaluated the accuracy and reliability of DL models for predicting third molar extraction difficulty and inferior alveolar nerve (IAN) injury risk.

**Methods:**

A systematic search was conducted across PubMed, Scopus, Web of Science, and Embase until September 2024, focusing on studies assessing DL models for predicting extraction complexity and IAN injury using PR images. The inclusion criteria required studies to report predictive performance metrics. Study selection, data extraction, and quality assessment were independently performed by two authors using the PRISMA and QUADAS-2 guidelines.

**Results:**

Six studies involving 12,419 PR images met the inclusion criteria. DL models demonstrated high accuracy in predicting extraction difficulty (up to 96%) and IAN injury (up to 92.9%), with notable sensitivity (up to 97.5%) for specific classifications, such as horizontal impactions. Geographically, three studies originated in South Korea and one each from Turkey and Thailand, limiting generalizability. Despite high accuracy, demographic data were sparsely reported, with only two studies providing patient sex distribution.

**Conclusion:**

DL models show promise in improving the preoperative assessment of third molar extraction. However, further validation in diverse populations and integration with clinical workflows are necessary to establish its real-world utility, as limitations such as limited generalizability, potential selection bias and lack of long-term follow up remain challenges.

## 1 Introduction

Third molar or wisdom tooth extraction is a common dental procedure often accompanied by varying degrees of complications. Studies highlight that complications can range from pain and swelling to more severe outcomes, such as mandibular fractures and nerve damage, emphasizing the need for meticulous surgical techniques and patient-specific risk assessments (1, 2). The prevalence of such complications underlines the importance of careful preoperative planning and radiographic evaluation to prevent complications such as tooth displacement into the submandibular space (3).

Therefore, predicting surgical difficulty in third molar extractions is critical for ensuring optimal surgical planning and effective patient management. Accurate preoperative assessments of complexity and potential risks enable dental surgeons to better manage procedural time, anticipate complications, and refine patient counseling (4, 5). Tools such as the Lambade-Dawane-Mali index further assist in aligning operative steps with case complexity, minimizing time, and effectively managing resources (6).

Panoramic radiography (PR) is a critical diagnostic tool in dental practice for assessing the third molars and evaluating their impact on adjacent anatomical structures. It enables clinicians to visualize the orientation, eruption level, and impaction pattern of third molars, which are essential for presurgical planning (7, 8). Although comparisons with cone-beam computed tomography (CBCT) show that CBCT offers higher precision, PR remains valuable for routine monitoring because of its accessibility and efficiency, capturing broad anatomical contexts that facilitate early intervention strategies.

Recent advancements in deep learning have significantly transformed medical imaging and diagnostics, thereby enhancing the accuracy and efficiency of disease detection and patient care. Innovations, such as segmentation models and AI-driven image communication systems, are improving data transmission in telemedicine and real-time diagnosis (9, 10). These advancements underscore the growing role of deep learning in creating data-driven, patient-centric healthcare solutions, highlighting the potential of deep learning models to enhance the prediction of surgical complexities and risks associated with third molar extraction.

The objective of our systematic review was to assess the predictive accuracy of deep learning models for determining the extraction difficulty of third molars and the risk of alveolar nerve injury. We followed PRISMA guidelines and applied inclusion criteria aligned with the study's objectives. We evaluated model performance across multiple studies to understand the reliability and clinical utility of these models in assessing third molar extraction complexity and potential nerve injury from panoramic radiographic images.

## 2 Methods

We conducted this systematic review following the Preferred Reporting Items for Systematic Review and Meta-Analysis (PRISMA) guidelines (11).

### 2.1 Search strategy and eligibility criteria

A comprehensive search was performed across PubMed, Scopus, Web of Science, and Embase from their inception up to September 2024. The search terms included: (((“machine learning” OR “ML” OR “deep learning” OR “DL” OR “neural network” OR “AI” OR “Artificial intelligence” OR “ANN” OR “Automated” OR “deep neural network” OR “DNN”) AND (“alveolar nerve injury” OR “mandibular nerve” OR ((extraction OR removal OR surgical) AND (difficulty OR complication))) AND (“wisdom Tooth” OR “wisdom teeth” OR “third molar”)). No restrictions were applied concerning language or publication date. Additionally, we manually searched the reference lists of relevant original studies and review articles to ensure a thorough search.

We included studies that: (1) evaluated the predictive accuracy of deep learning models, (2) assessed the prediction of extraction difficulty of third molar or the alveolar nerve injury from panoramic radiographic images, and (3) reported specific performance metrics related to the predictive accuracy of the models. Only studies that provided these performance metrics were considered. Studies that did not meet all of these inclusion criteria, reported other types of metrics but not predictive performance were excluded from the review.

### 2.2 Study selection and data extraction

Two authors independently screened the titles and abstracts of all identified studies using the predefined eligibility criteria. Full texts of potentially relevant studies were further reviewed in detail by the same authors. Any discrepancies between the two reviewers were resolved by consulting a third author, ensuring consensus. Data were independently extracted by the same reviewers using an Excel sheet. Extracted information included study characteristics (e.g., first author, publication year, country, and study design), baseline patient data (age, gender, and sample size), as well as sensitivity and specificity of each machine learning model's predictive accuracy.

### 2.3 Risk of bias assessment

The methodological quality of the included studies was independently assessed by two authors using the Quality Assessment of Diagnostic Accuracy Studies, Version 2 (QUADAS-2) tool (12). This tool evaluates four domains: (1) patient selection, (2) index test, (3) reference standard, and (4) flow and timing. Each domain was assessed for both the risk of bias and the applicability of the first three domains. The overall risk of bias for each study was categorized as low, some concern, or high.

## 3 Results

### 3.1 Search results

**Figure 1:**
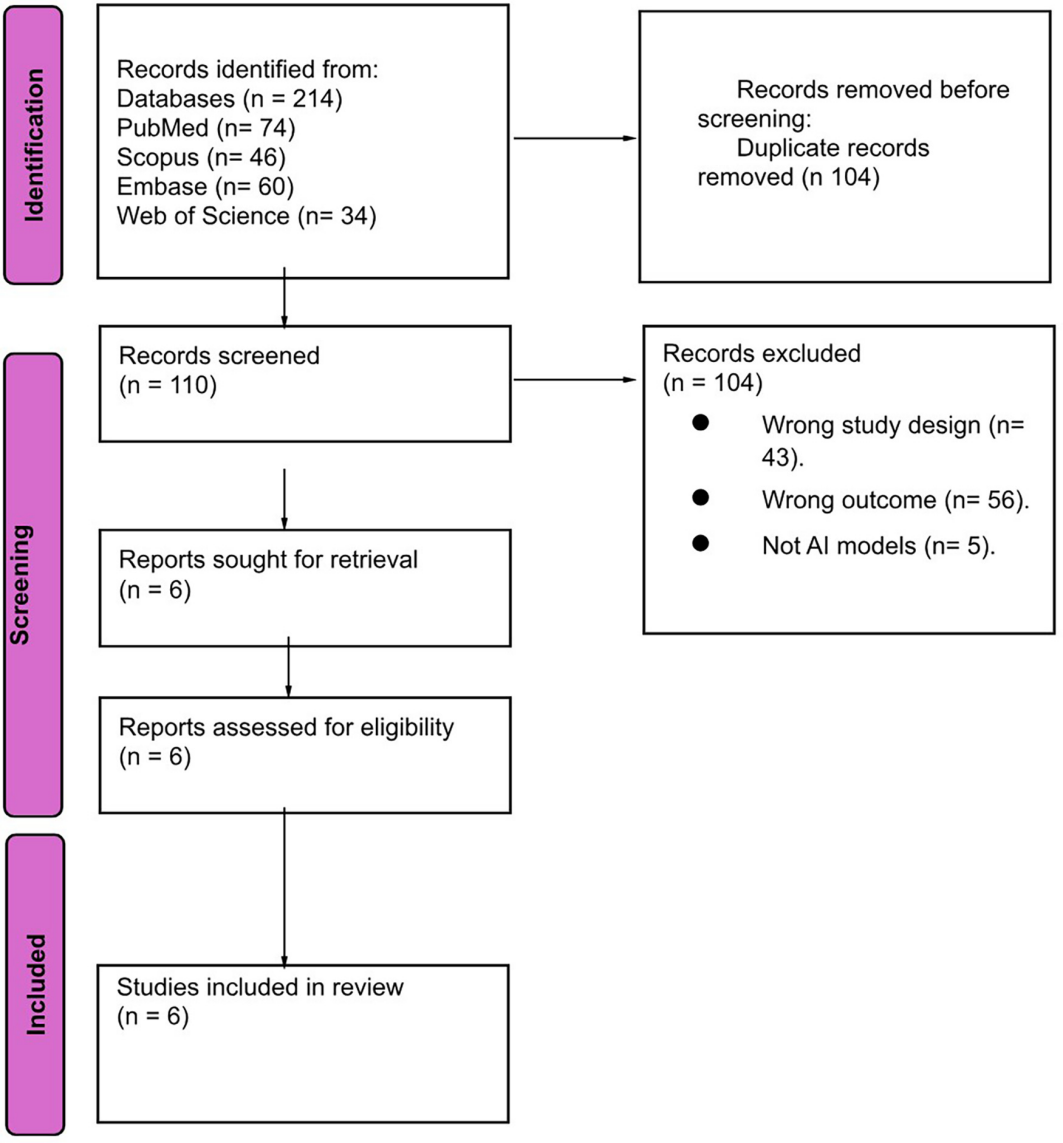
Flowchart for included studies.

We obtained 214 papers from four electronic databases. Using EndNote software, 104 duplicate papers were removed. After screening by title and abstract, 104 papers were excluded. We evaluated the full texts of the remaining six studies for final eligibility. Finally, six papers were included in our review. The study selection process is shown in Figure 1.

### 3.2 Summary of the included studies

Six studies were included in our systematic review (13–17). They were conducted between 2021 and 2024. 12,419 panoramic radiographic images were analyzed across the included studies. Out of six studies. Three studies assessed the role of deep learning in predicting difficulty, two studies reported the role of deep learning in predicting alveolar nerve injury, and one study evaluated both. Four studies reported the number of extracted third molars assessed which was 11,190. It is important to note that the discrepancy arises from that some studies may have analyzed multiple radiographs per patient or assessed different tooth types in the panoramic radiographic images which were not always linked to the exact number of extraction. 12,419 images were analyzed while 11,190 were assessed. Therefore, the total number of images analyzed exceed the number of extracted third molar assessed. Only two studies reported the sex of participants which was 313 males and 312 females. Five studies reported the origin of the included patients: three studies conducted in South Korea, one study conducted in Turkey and one study conducted in Thailand. Full details about the characteristics of the included studies reported in Table 1.

**Table 1:** Characteristics of the included studies.

Study/Year	Study type	Country	Number of patients	Mean age	Number mandibular third molars	Model	Aim	Conclusion
Yoo et al. 2021 (17)	Cohort	South Korea	305 males and 295 females	27.5	1,053 from 600 preoperative panoramic adiographic images	CNN -based DL model (ResNet-34)	This paper proposes a convolutional neural network (CNN)-based deep learning model for predicting and evaluating he difficulty of extracting a mandibular third molar using a panoramic radiographic image.	The results confirm that the proposed CNN-based deep learning model could be used to predict the the difficulty of extracting a mandibular third molar using a panoramic radiographic image.
Picoli et al. 2023 (16)	Controlled Trial	/	8 males and 17 females	27	50 were surgically removed in 25 patients and 25 panoramic radiographic images	3D-AI DL model based on CNN- Virtual Patient Creator	To compare a three-dimensional (3D) artificial intelligence (AI)- driven model with panoramic radiography (PANO) and cone-beam computed tomography (CBCT) in assessing the risk of the inferior alveolar nerve (IAN) injury after removal of the mandibular wisdom tooth (M3M) through a within-patient controlled trial.	This within-patient controlled trial study revealed that risk assessment for IAN injury after MM3 removal was rather similar for 3D-AI, PANO, and CBCT, with a sensitivity for injury prediction reaching up to 0.87 for 3D-AI and 0.89 for CBCT.
Lee et al. 2022 (15)	Cohort	South Korea	/	/	8,720 from 4,903 panoramic radiographic images	R50+ViT-L/32 Vision Transformer hybrid DL model (deep neural network) model	We propose a method of automatically detecting mandibular third molars in the panoramic radiographic images and predicting the extraction difficulty and likelihood of inferior alveolar nerve (IAN) injury.	This study demonstrates that a deep neural network can predict both the extraction the difficulty of mandibular third molars and the likelihood of IAN injury following extraction in panoramic radiographic images.
Trachoo et al. 2024 (14)	Cohort	Thailand	784	/	1,367 from 784 panoramic radiographic images	Computer-aided visualisation−based DL system- multiclass image classification vision Transformers (ViT)	The aim of this study was to develop and evaluate a computer-aided visualisation−based deep learning (DL) system using a panoramic radiograph to predict the difficulty level of surgical removal of an impacted LM3.	The development of a 3-phase computer-aided visualization-based DL system has yielded very good performance in using panoramic radiographs to predict the difficulty level of surgically removing an impacted MM3.
Torul et al. 2024 (4)	Cohort	Turkey	/	/	708 radiographs having at least one impacted maxillary third molar tooth were evaluated in this study.	CNN- based DL model (YOLOv5x)	The aim of this study is to determine if a deep learning (DL) model can predict the surgical difficulty for impacted maxillary third molar tooth using panoramic images before surgery.	The results showed that the proposed DL model could be effective for predicting the surgical difficulty of an impacted maxillary third molar tooth using panoramic radiographs, and this approach might help as a decision support mechanism for the clinicians in the peri-surgical period.
Gong et al. 2024 (13)	Article	South Korea	/	/	5,374 panoramic radiographic images	CNN DL-based classification model (CD-IAN injury class)	To propose an automated system based on panoramic radiographs, featuring a novel segmentation model SS-TransUnet and classification algorithm CD-IAN injury class.	Our classification algorithm achieved an accuracy of 0.846, surpassing deep learning-based models by 3.8%, confirming the effectiveness of our system.

### 3.3 Systematic review

#### 3.3.1 Deep learning models in predicting the extraction difficulty of third molar

Four studies assessed the role of deep learning in extraction difficulty of third molar Table 2. Different definitions of the difficulty index definition were used across the included studied, all the definitions reported in Supplementary Table S1. Yoo et al. (17) classified the difficulty of extraction to three levels, the highest accuracy (90.23) of the model was achieved in C3 which was when the occlusal surface of the mandibular third molar was compared with the distal surface of the mandibular second molar. Also, the highest sensitivity (94.84%) was achieved in C3 score 3 which was when they were close to perpendicular. However, the highest specificity (97.65%) was achieved in C3 score 2 which was when they were close to parallel.

**Table 2:** Summery of four studies assessed the role of deep learning in extraction difficulty of third molar.

Study	Difficulty index/score	Difficulty criterion	Score/Level	AUC	Accuracy	Precision	F1 score	Sensitivity (Recall)	Specificity
Yoo et al.	Pederson difficulty index	C1 (Depth)	1	–	78.91%	–	–	88.13%	92.05%
			2					72.77%	84.12%
			3					78.63%	90.89%
		C2 (Ramal relationship)	1	–	82.03%	–	–	71.69%	94.22%
			2					90.73%	69.52%
			3					61.36%	98.29%
		C3 (Angulation)	1	–	90.23%	–	–	94.15%	92.67%
			2					89.53%	97.65%
			3					94.84%	95.24%
			4					0%	100%
Lee et al.	Score based Pell and Gregory's classification & Winter's classification	VE	1	98%	83.5%	–	66.35%	–	–
		STI	2	91%				–	–
		PBI	3	91%				–	–
		CBI	4	91%				–	–
Trachoo et al.	Pederson difficulty index	Minimally difficult (novice)	1	–	89.78%	83.33%	87.72%	92.59%	87.95%
		Moderately difficult (intermediate)	2	–	85.4%	87.14%	85.91%	84.72%	86.15%
		Very difficult (expert)	3	–	95.62%	85.71%	66.67%	54.55%	99.21%
Torula et al.	Angulation (H)	H-multiclass detection	–	–	–	69.64%	81.25%	97.50%	–
	Relationwithramus (R).	R-multiclass detection	–	–	–	70.43%	81%	95.29%	–
	Depth (V)	V-multiclass detection	–	–	–	68.85%	80.38%	96.55%	–
	Relation with maxillarysinus(S)	S- multiclass detection	–	–	–	61.94%	73.29%	89.74%	–

Lee et al. and Trachoo et al. classified the extraction difficulty into classes by the surgeon's perspective (14, 15). In lee et al. study, the highest accuracy of the model was achieved in first class when a simple extraction was done without gum incision or bone fracture. Additionally, it is worth noting that the model continued to achieve a high accuracy of 91% even when applied in different scenarios, such as when extraction was performed following a gum incision, when tooth segmentation was necessary, or in cases where more than two-thirds of the crown was impacted. However, Trachoo et al. classified the extraction difficulty into three classes (4). The model achieved 96% accuracy for both novice and experts' level and 85% accuracy for intermediate level. Alternatively, Torul et al. built a multiclass detection system related to horizontal positions, relation with ramus, relation with sinus and vertical positions. The highest sensitivity (97.5%) was achieved in detecting horizontal positions.

#### 3.3.2 Deep learning models in predicting the alveolar nerve injury

Three studies assessed the role of deep learning in predicting alveolar nerve injury. The definitions of the alveolar nerve injury reported in Supplementary Table S1. Picoli et al. built a deep learning model to predict any nerve injury, the model achieved 73% sensitivity and 41% specificity with a 57% AUC (16). Gong et al. and Lee et al. classified the nerve injury to three levels (13–15). Lee et al. (15) showed that the model achieved the highest AUC (94%) in predicting N3 which was when the mandibular third molar interrupts two lines of the IAN canal in the panoramic radiographic image. Gong et al. (13) assessed the model in tooth 38 and 48. The model showed high sensitivity in predicting the N2 for booth tooth 38 (92.9%) and tooth 48 (93.1%), which was when the mandibular third molar interrupts one line of the IAN canal in the panoramic radiographic image. The reported performance of deep learning models in predicting the alveolar nerve injury in these studies is summarized in Table 3.

**Table 3:** The performance of deep learning models in predicting the alveolar nerve injury.

Study	Model	AUC	Precision	Sensitivity (Recall)	Specificity	F1-Score	Accuracy
Picoli et al.	3D-AI Model based on convolutional neural network- Virtual Patient Creator	57%	–	73%	41%	–	–
Lee et al.	R50+ViT-L/32 Vision Transformer deep neural network model	N1: 91%	–	–	–	75.55%	81.1%
		N2: 86%	–				
		N3: 94%	–				
Gong et al.	Convulotional neural network DL-based classification model (CD-IAN injury class) (38)	–	N1: 75.0%	N1: 80.2%	–	N1:77.5%	84.7%
		–	N2: 86.1%	N2: 92.9%	–	N2: 89.4%	
		–	N3: 92.6%	N3: 49.5%	–	N3: 64.5%	
	Convulotional neural network DL-based classification model (CD-IAN injury class) (48)	–	N1:74.8%	N1:77.6%	–	N1:76.1%	84.7%
		–	N2:85.6%	N2:93.1%	–	N2:89.2%	
		–	N3:91.2%	N3:54.4%	–	N3:68.1%	

### 3.4 Quality assessment

QUADAS-2 quality assessment tool was used to assess the quality of the included studies. Two studies were had a low risk of bias in all assessed domains. Two studies had unclear risk of bias in reference standard and index test assessment. Also, one study was classified to have unclear risk of bias in index test alone and another one in reference standard domain alone. Full information about the quality assessment is reported in Supplementary Figure S1.

## 4 Discussion

This systematic review synthesized evidence from six studies evaluating deep learning (DL) models for predicting extraction difficulty of third molars and the risk of inferior alveolar nerve (IAN) injury from panoramic radiographic images (13–17). These studies employed various deep learning architectures based convolutional neural networks (CNN), achieving overall high accuracy and sensitivity in predicting extraction difficulty (up to 96%) and IAN injury (up to 92.9%). Notably, Yoo et al. (2021) reported the highest accuracy in angulation classification for mandibular extractions using the Pederson difficulty score (17), while Gong et al. (2024) observed high sensitivity in IAN injury classification (13).

The findings align with prior research on AI-driven models in dental imaging, showing that DL models can surpass traditional methods in objective and rapid assessment of complex clinical scenarios (18–27). Traditional indices, such as the Pederson Difficulty Score, rely on clinician interpretation, which may be subject to variability and bias. In contrast, DL models have shown to automate and standardize risk assessments with high inter-rater reliability. For example, Lee et al. (2022) achieved accuracy levels exceeding 80% across both difficulty and IAN injury predictions, demonstrating that DL algorithms can enhance consistency in diagnostic outcomes across different patient cases (15).

The integration of DL models into clinical practice for preoperative assessment has the potential to significantly improve patient outcomes and resource management (27). By accurately predicting extraction difficulty and IAN injury risk, DL tools could assist surgeons in developing patient-specific treatment plans, potentially minimizing surgical time and complication rates. These models are especially valuable for less experienced clinicians, providing a decision-support mechanism that reinforces their diagnostic judgment (15). Additionally, as demonstrated by Picoli et al. (2023), AI models offer comparable sensitivity to CBCT in assessing IAN injury risk, suggesting that panoramic imaging alone could serve as an effective, lower-cost alternative in certain cases (16).

Recent studies on IAN block injections have further contextualized the importance of precision in medical procedures. These studies highlight the challenges of managing complications and ensuring their effectiveness (28, 29).

The included studies provided substantial insights but varied widely in their sample sizes, imaging protocols, and model architectures, potentially affecting generalizability. These inconsistencies in methods and reported results may introduce bias and limit the reliability of the overall conclusion. Differences in sample sizes and imaging protocols could result in varying model performance across studies, making a consistent standard for DL-based diagnostic tools difficult. Most studies reported high accuracy and sensitivity metrics; however, performance variability was noted depending on the complexity of anatomical structures, such as in IAN proximity classifications. Furthermore, only two studies reported patient demographics, and none accounted for the potential influence of these factors on model performance. The limited diversity in patient populations, with the majority being from specific regions such as South Korea and Turkey, may impact the model's transferability to other clinical settings (14, 16).

This review has several limitations. First, the limited number of studies included may result in publication bias, as studies with positive results are more likely to be published. Additionally, variability in the methods used for difficulty and injury prediction limited the possibility of meta-analysis, and the lack of standardized reporting on demographic factors restricts the applicability of findings across broader patient populations. Furthermore, while DL models achieved promising accuracy, real-world deployment would require extensive validation and recalibration on diverse datasets, which was not covered in the studies reviewed. Model should be validated on diverse external database and recalibrated using parameters tailored to demographic and clinical variations. Techniques like transfer learning of domain adaptation can further enhance performance and generalizability in real-word settings. Additionally, only two studies reported the sex of participants, this limited the ability to assess the potential impact of demographic factors, such as sex, on the performance of the models. Also, the geographic distribution of the studies may limit the generalizability of our finding to other populations.

## 5 Conclusion

This systematic review provides strong evidence that DL models significantly enhanced the prediction accuracy of third molar extraction difficulties and IAN injury risks using panoramic radiographic images. These models outperform traditional methods and offer more precise and reliable preoperative assessments demonstrating their potential for clinical use that could reduce surgical complications and improve patient outcomes. However, the studies reviewed showed variability in methods and samples, making broader validation essential across diverse populations and geographic locations to ensure global applicability. Future research should focus on standardizing DL model testing, developing clinical guidelines, and integrating these tools with other diagnostic technologies to further refine their effectiveness in the clinical setting.

Future research should aim to standardize DL model development and testing protocols in dental imaging to improve reproducibility and generalizability across diverse patient populations. Further studies are needed to address demographic factors such as gender and geographic factors, enabling results to be generalized and widely applicable. Large-scale, multi-center prospective studies with diverse patient demographics are needed to validate these models' real-world utility. Exploring the integration of CBCT alongside panoramic images within DL models could enhance diagnostic accuracy, especially in complex cases with high-risk nerve proximity. Additionally, research into explainable AI could improve clinical adoption by enhancing model interpretability, addressing the current “black-box” challenge in DL applications in medicine. Finally, studies should investigate ethical, legal, and patient privacy considerations to ensure safe and equitable AI deployment in clinical practice (4, 13).

## Data Availability

The original contributions presented in the study are included in the article/Supplementary Material, further inquiries can be directed to the corresponding author.
