# Supplementary material for: Predicting alveolar nerve injury and the difficulty level of extraction impacted third molars: a systematic review of deep learning approaches
Source: Front Dent Med. 2025 May 20;6:1534406. doi: 10.3389/fdmed.2025.1534406 (PMC12129997; doi:10.3389/fdmed.2025.1534406)
Supplement: Supplementary file 1 [file Table1.docx]

| **Study** | **RISK OF BIAS** | | | | **APPLICABILITY CONCERNS** | | |
| --- | --- | --- | --- | --- | --- | --- | --- |
|  | **PATIENT SELECTION** | **INDEX TEST** | **REFERENCE STANDARD** | **FLOW AND TIMING** | **PATIENT SELECTION** | **INDEX TEST** | **REFERENCE STANDARD** |
| Yoo et al. | \| ☺ \| ☺ \| ☺ \| ☺ \| ☺ \| ☺ \| ☺ \| \| --- \| --- \| --- \| --- \| --- \| --- \| --- \| | \| ☺ \| ☺ \| ☺ \| ☺ \| ☺ \| ☺ \| ☺ \| \| --- \| --- \| --- \| --- \| --- \| --- \| --- \| | \| ☺ \| ☺ \| ☺ \| ☺ \| ☺ \| ☺ \| ☺ \| \| --- \| --- \| --- \| --- \| --- \| --- \| --- \| | \| ☺ \| ☺ \| ☺ \| ☺ \| ☺ \| ☺ \| ☺ \| \| --- \| --- \| --- \| --- \| --- \| --- \| --- \| | \| ☺ \| ☺ \| ☺ \| ☺ \| ☺ \| ☺ \| ☺ \| \| --- \| --- \| --- \| --- \| --- \| --- \| --- \| | \| ☺ \| ☺ \| ☺ \| ☺ \| ☺ \| ☺ \| ☺ \| \| --- \| --- \| --- \| --- \| --- \| --- \| --- \| | \| ☺ \| ☺ \| ☺ \| ☺ \| ☺ \| ☺ \| ☺ \| \| --- \| --- \| --- \| --- \| --- \| --- \| --- \| |
| Picoli et al. | \| ☺ \| ☺ \| ☺ \| ☺ \| ☺ \| ☺ \| ☺ \| \| --- \| --- \| --- \| --- \| --- \| --- \| --- \| | \| ☺ \| ☺ \| ☺ \| ☺ \| ☺ \| ☺ \| ☺ \| \| --- \| --- \| --- \| --- \| --- \| --- \| --- \| | \| ? \| ☺ \| ☺ \| ☺ \| ☺ \| ☺ \| ☺ \| \| --- \| --- \| --- \| --- \| --- \| --- \| --- \| | \| ☺ \| ☺ \| ☺ \| ☺ \| ☺ \| ☺ \| ☺ \| \| --- \| --- \| --- \| --- \| --- \| --- \| --- \| | \| ☺ \| ☺ \| ☺ \| ☺ \| ☺ \| ☺ \| ☺ \| \| --- \| --- \| --- \| --- \| --- \| --- \| --- \| | \| ? \| ☺ \| ☺ \| ☺ \| ☺ \| ☺ \| ☺ \| \| --- \| --- \| --- \| --- \| --- \| --- \| --- \| | \| ☺ \| ☺ \| ☺ \| ☺ \| ☺ \| ☺ \| ☺ \| \| --- \| --- \| --- \| --- \| --- \| --- \| --- \| |
| Lee et al. | \| ☺ \| ☺ \| ? \| ☺ \| ☺ \| ☺ \| ☺ \| \| --- \| --- \| --- \| --- \| --- \| --- \| --- \| | \| ? \| ☺ \| ? \| ☺ \| ☺ \| ☺ \| ☺ \| \| --- \| --- \| --- \| --- \| --- \| --- \| --- \| | \| ☺ \| ☺ \| ? \| ☺ \| ☺ \| ☺ \| ☺ \| \| --- \| --- \| --- \| --- \| --- \| --- \| --- \| | \| ☺ \| ☺ \| ? \| ☺ \| ☺ \| ☺ \| ☺ \| \| --- \| --- \| --- \| --- \| --- \| --- \| --- \| | \| ☺ \| ☺ \| ? \| ☺ \| ☺ \| ☺ \| ☺ \| \| --- \| --- \| --- \| --- \| --- \| --- \| --- \| | \| ☺ \| ☺ \| ? \| ☺ \| ☺ \| ☺ \| ☺ \| \| --- \| --- \| --- \| --- \| --- \| --- \| --- \| | \| ☺ \| ☺ \| ? \| ☺ \| ☺ \| ☺ \| ☺ \| \| --- \| --- \| --- \| --- \| --- \| --- \| --- \| |
| Trachoo et al. | \| ☺ \| ☺ \| ☺ \| ☺ \| ☺ \| ☺ \| ☺ \| \| --- \| --- \| --- \| --- \| --- \| --- \| --- \| | \| ☺ \| ☺ \| ☺ \| ☺ \| ☺ \| ☺ \| ☺ \| \| --- \| --- \| --- \| --- \| --- \| --- \| --- \| | \| ☺ \| ☺ \| ☺ \| ☺ \| ☺ \| ☺ \| ☺ \| \| --- \| --- \| --- \| --- \| --- \| --- \| --- \| | \| ☺ \| ☺ \| ☺ \| ☺ \| ☺ \| ☺ \| ☺ \| \| --- \| --- \| --- \| --- \| --- \| --- \| --- \| | \| ☺ \| ☺ \| ☺ \| ☺ \| ☺ \| ☺ \| ☺ \| \| --- \| --- \| --- \| --- \| --- \| --- \| --- \| | \| ? \| ☺ \| ☺ \| ☺ \| ☺ \| ☺ \| ☺ \| \| --- \| --- \| --- \| --- \| --- \| --- \| --- \| | \| ☺ \| ☺ \| ☺ \| ☺ \| ☺ \| ☺ \| ☺ \| \| --- \| --- \| --- \| --- \| --- \| --- \| --- \| |
| Torula et al. | \| ☺ \| ☺ \| ☺ \| ☺ \| ☺ \| ☺ \| ☺ \| \| --- \| --- \| --- \| --- \| --- \| --- \| --- \| | \| ☺ \| ☺ \| ☺ \| ☺ \| ☺ \| ☺ \| ☺ \| \| --- \| --- \| --- \| --- \| --- \| --- \| --- \| | \| ☺ \| ☺ \| ☺ \| ☺ \| ☺ \| ☺ \| ☺ \| \| --- \| --- \| --- \| --- \| --- \| --- \| --- \| | \| ☺ \| ☺ \| ☺ \| ☺ \| ☺ \| ☺ \| ☺ \| \| --- \| --- \| --- \| --- \| --- \| --- \| --- \| | \| ☺ \| ☺ \| ☺ \| ☺ \| ☺ \| ☺ \| ☺ \| \| --- \| --- \| --- \| --- \| --- \| --- \| --- \| | \| ☺ \| ☺ \| ☺ \| ☺ \| ☺ \| ☺ \| ☺ \| \| --- \| --- \| --- \| --- \| --- \| --- \| --- \| | \| ☺ \| ☺ \| ☺ \| ☺ \| ☺ \| ☺ \| ☺ \| \| --- \| --- \| --- \| --- \| --- \| --- \| --- \| |
| Gong et al. | \| ☺ \| ☺ \| ☺ \| ☺ \| ☺ \| ☺ \| ☺ \| \| --- \| --- \| --- \| --- \| --- \| --- \| --- \| | \| ☺ \| ☺ \| ☺ \| ☺ \| ☺ \| ☺ \| ☺ \| \| --- \| --- \| --- \| --- \| --- \| --- \| --- \| | \| ? \| ☺ \| ☺ \| ☺ \| ☺ \| ☺ \| ☺ \| \| --- \| --- \| --- \| --- \| --- \| --- \| --- \| | \| ☺ \| ☺ \| ☺ \| ☺ \| ☺ \| ☺ \| ☺ \| \| --- \| --- \| --- \| --- \| --- \| --- \| --- \| | \| ☺ \| ☺ \| ☺ \| ☺ \| ☺ \| ☺ \| ☺ \| \| --- \| --- \| --- \| --- \| --- \| --- \| --- \| | \| ? \| ☺ \| ☺ \| ☺ \| ☺ \| ☺ \| ☺ \| \| --- \| --- \| --- \| --- \| --- \| --- \| --- \| | \| ☺ \| ☺ \| ☺ \| ☺ \| ☺ \| ☺ \| ☺ \| \| --- \| --- \| --- \| --- \| --- \| --- \| --- \| |
|  |  |  |  |  |  |  |  |

☺Low Risk ☹High Risk ? Unclear Risk

**Figure S1:** A quality assessment of included studies.
