# Supplementary material for: Predicting alveolar nerve injury and the difficulty level of extraction impacted third molars: a systematic review of deep learning approaches
Source: Front Dent Med. 2025 May 20;6:1534406. doi: 10.3389/fdmed.2025.1534406 (PMC12129997; doi:10.3389/fdmed.2025.1534406)
Supplement: Supplementary file 2 [file Table2.docx]

|  | |  |
| --- | --- | --- |
| Supplements | |  |
| Yoo et al. | C1 (Depth) | Te midpoint of an occlusal surface of the impacted third molar was set as the evaluation point. When the evaluation point was above the occlusal surface of the mandibular second molar, we recorded the score as a 1, and when it was below, we recorded it as a 2. When the entire tooth was below the occlusal surface of the mandibular second molar, we recorded the score as a 3. |
|  | C2 (Ramal Relationship) | In mesio-angulation and horizontal angulation cases, a contact point of the man- dibular third molar and the mandibular ramus was set as the evaluation point. Te evaluation point was  compared with the distal point of the cemento-enamel junction of the mandibular third molar. When the contact point was disto-apical, we recorded the score as a 1. When the contact point was mesio-occlusal, we recorded it as a 2. In the vertical and distoangular cases, we used the same points but only considered the occluso-apical position. When the contact point was apical, we recorded it as a 1. When the contact point was occlusal, we recorded it as a 2. Tose scores for cases in which the entire crown was impacted were recorded as a 3. |
|  | C3 (Angulation) | Te occlusal surface of the mandibular third molar was compared with the distal surface of the mandibular second molar. When they were close to perpendicular, we recorded the score as a 3, and when they were close to parallel, we recorded the score as a 2; otherwise, we recorded the score as a 1. Finally, we scored those cases with an angle of below 90° as a 4. |
| Lee et al. | N1 (low) | The mandibular third molar does not reach the IAN canal in the panoramic radiographic image. |
|  | N2 (medium) | The mandibular third molar interrupts one line of the IAN canal in the panoramic radiographic image. |
|  | N3 (high) | The mandibular third molar interrupts two lines of the IAN canal in the panoramic radiographic image. |
| Lee et al. | Vertical Eruption (VE) | Simple extraction without gum incision or bone fracture. |
|  | Soft tissue impaction (STI) | Extraction after a gum incision. |
|  | Partial bony impaction (PBI) | Tooth segmentation is required for extraction. |
|  | Complete bony impaction (CBI) | Where more than two-thirds of the crown is impacted, this requires tooth segmentation and bone fracture. |
| Trachoo et al. | Novice | ViT was adopted to develop the multiclass image classification model to predict 3 difficulty levels of surgically removing impacted LM3s: novice, intermediate, and expert. ViT. |
|  | Intermediate |  |
|  | Expert |  |
| Torula et al. | H-multiclass detection | Horizontal position (H):  H1: Vertical-1 H2: Distoangular-2 H3: Mesioangular-3 H4: Horizontal-4 H5: Other-5 |
|  | R-multiclass detection | Relation with ramus (R):  R0: There is sufficient space between the ramus and the distal part of the second molar to accommodate the mesiodistal diameter of the third molar-0,  R1: The space between the second molar and the ramus is less than the mesiodistal diameter of the third molar-1,  R2: All or most of the third molar is within the ramus-2. |
|  | V-multiclass detection | Vertical position (V):  VA: The occlusal surface of the impacted tooth is approximately at the same level as the occlusal surface of the second molar-1  VB: The occlusal surface of the impacted tooth is in the middle of the crown of the adjacent second molar tooth-2  VC: Occlusal surface of the crown of the impacted tooth, below the cervical line of the adjacent molar or even deeper-3.) |
|  | S- multiclass detection | Relation with sinus (S):  S0: No bone or thin bone section between impacted maxillary third molar and maxillary sinus; Any part of the maxillary third molar is in contact with the sinus wall/floor or beyond the sinus floor-0,  S1: There is ≥ 2 mm of bone between the impacted maxillary third molar and the maxillary sinus-1. |
| Gong et al. | N1 | The mandibular third molar does not reach the IAN canal in the panoramic radiographic image. |
|  | N2 | The mandibular third molar interrupts one line of the IAN canal in the panoramic radiographic image. |
|  | N3 | The mandibular third molar interrupts two lines of the IAN canal in the panoramic radiographic image. |

**Table 1S:** Various definitions of the alveolar nerve injury as mentioned in different studies.
